# What Provider Frequently Asked Questions Miss: Evaluating Unmet Attention-Deficit/Hyperactivity Disorder Information Needs Through Comparison of Online Community Posts Using Large Language Model–Assisted Semantic Analysis in a Mixed Methods Study

**DOI:** 10.2196/96060

**Published:** 2026-09-11

**Authors:** Jaeeun Baek, Hyeoneui Kim

**Affiliations:** 1 Healthcare AI Research Institute, Seoul National University Hospital, Seoul, Republic of Korea; 2 College of Nursing, Seoul National University, 103 Daehak-ro, Jongno-gu, Seoul, 03080, Republic of Korea, 82 02-740-8483; 3 Research Institute of Nursing Science, Jongno-gu, Republic of Korea

**Keywords:** attention-deficit/hyperactivity disorder, ADHD, unmet information needs, online communities, frequently asked questions, large language models, semantic analysis

## Abstract

**Background:**

Attention-deficit/hyperactivity disorder (ADHD) is a prevalent neurodevelopmental disorder that affects the functioning and quality of life of individuals throughout their lifespan. Despite the extensive information available online, patients and caregivers continue to report unmet needs, particularly regarding diagnosis, treatment, medication effects, comorbidities, and long-term management strategies. Existing provider-generated frequently asked questions (FAQs) are widely used, but often fail to fully capture the concerns expressed in online communities.

**Objective:**

This study aimed to (1) evaluate the extent to which provider-generated ADHD FAQs cover questions from online communities, (2) identify unmet information needs by analyzing questions with low semantic similarity to FAQs, and (3) compare the response styles of provider-generated answers with community-generated answers through large language model (LLM)–assisted analysis.

**Methods:**

ADHD-related questions from a Korean online community were semantically compared with provider-generated FAQs using sentence embedding–based similarity analysis to assess coverage and identify matched versus unmatched questions. Unmatched questions underwent topic modeling using the LimTopic framework, integrating BERTopic with LLM-assisted summarization to uncover unmet needs. An LLM-assisted content analysis was conducted on the answers to the high-similarity FAQ–community question pairs, enabling an examination of the response styles used by each group when addressing the public.

**Results:**

Through similarity comparison using embedding models and manual verification, the paraphrase-multilingual-MiniLM-L12-v2 (MBERT) model, which achieved the highest *F*_1_-score of 0.45, was selected as the final embedding model. The optimal similarity threshold determined for this model was 0.766, and the coverage of questions with similarity above this threshold between FAQs and the online community was 52.09% (2598/4988). Most of the coverage was concentrated on 18 FAQs. Online community questions below the similarity threshold were reviewed by experts after LimTopic analysis, resulting in the identification of 12 categories of unmet consumer needs, including school and social support, treatment accessibility, psychological support, and comorbidity management. Response style analysis revealed significant differences between evidence and authority signaling and the actionability dimension.

**Conclusions:**

Provider-generated ADHD FAQs covered approximately half of consumers’ questions, revealing substantial gaps in information provision for patients with ADHD. Health information on ADHD should expand beyond basic medical knowledge to address consumers’ real-world experiences, including access to care, school and social support, evidence-based treatments, daily functioning strategies, psychological support, health care navigation, and comorbidity management. Integrating these elements into provider-generated FAQs can create more comprehensive, consumer-centered resources that better support informed decision-making and long-term self-management.

## Introduction

Attention-deficit/hyperactivity disorder (ADHD) is a neurodevelopmental disorder characterized by persistent symptoms of inattention, hyperactivity, and impulsivity [1]. The 2023 National Mental Health Status Report identified behavioral disorders, including ADHD, as the most prevalent mental health issues among children and adolescents, comprising 46.7% of the reported cases [1]. Despite ongoing treatment, individuals with ADHD and their caregivers continue to experience substantial unmet information needs [2]. Parents of children with ADHD require accurate, accessible, comprehensible, and up-to-date information [3]. Such information is particularly critical for effective coping during the transition from adolescence to adult ADHD [4]. Therefore, effective management of ADHD requires timely access to accurate information to prevent disease progression and minimize the need for pharmacological intervention.

Parents play a pivotal role in managing pediatric disorders; however, insufficient knowledge about ADHD may result in maladaptive parenting practices, increasing the risk of symptom exacerbation or the emergence of comorbid conditions in affected children [5]. Although abundant ADHD-related information is available online, its quantity does not necessarily translate into user satisfaction or adequately meet informational needs [6]. An analysis of the information in the top 50 TikTok videos tagged with ADHD tests found that 92% provided inadequate information [7]. Conversely, limited research has examined whether information on ADHD disseminated by official sources adequately addresses stakeholders’ information needs. Moreover, while parents frequently seek guidance from health care providers, discrepancies may exist between providers’ perceptions of information priorities and those of patients and caregivers [8]. Furthermore, verbal information from health care professionals, while reliable, is focused on a clinical perspective [9]. While providers may prefer to present information as a list of frequently asked questions (FAQs), this approach often fails to engage or influence the target audience [10]. Responses that describe best practices alone are insufficient, as they do not adequately account for accessibility barriers, particularly constraints in accessing timely and coordinated ADHD services [11]. To develop information that adequately reflects the diverse and multifaceted needs of individuals, it is essential to comprehensively identify and characterize unmet information needs across various aspects [3,12]. Recognizing the information needs of both patients and caregivers is essential for delivering patient-centered care [13]. Failure to provide patients with sufficient information can lead to problems such as quality-of-life concerns, depression, poor parenting, and social stigma [5,6,14].

However, studies identifying the information needs of individuals with ADHD are rare. One study indicated that both parents and children prefer online health information, suggesting that effective strategies should integrate reputable websites with physicians providing ADHD information [15,16]. Most information needs are identified through interviews and include information needs regarding the treatment and prognosis of ADHD [17], information needs for the transition from adolescence to adulthood [18], information needs for parenting and medical treatment [19], and the need for improved health information on the internet [20,21]. While interviews are essential for identifying gaps in complex needs and tracking evolving patterns longitudinally, their high time and cost demands make them unscalable for repeated and large-scale data collection [22].

Algorithmic approaches using large-scale data offer a more efficient and generalizable solution for systematic need tracking and computational identification of information gaps, overcoming the scalability limitations of traditional methods [23]. The Sentence-Bidirectional Encoder Representations from Transformers (SBERT) model, when applied to assess the semantic similarity between consumer questions and answers, enhanced user satisfaction [24]. Furthermore, integrating machine learning with natural language processing to develop a FAQ chatbot improved both response accuracy and overall user satisfaction [25]. The integration of large language models (LLMs) with SBERT improves semantic similarity and enhances the accuracy of consumer query matching [26]. Building on these advances in computational text analysis and LLM-based approaches, this study examined the alignment between provider-generated ADHD FAQs from official Korean institutions and questions posted in an online community using semantic similarity analysis and LLM-assisted approaches to identify unmet public information needs. This study aimed to answer the following research questions (RQs):

- RQ1. To what extent do provider-generated ADHD FAQs provide semantic coverage of questions posted in online consumer communities regarding ADHD?
- RQ2. What topics from online community questions show low semantic similarity to provider-generated ADHD FAQs?
- RQ3. How do provider-generated FAQs responses differ from online community responses in terms of response style, based on exploratory LLM-assisted content analysis?

## Methods

### Data Sources

The data sources comprised 2 datasets: ADHD-related FAQs were obtained from publicly available content on the “ADHD Baroalgi (Understanding ADHD)” website operated by the Korean Medical Association [27] and the “National Center for Mental Health Portal” provided by the Ministry of Health and Welfare [28]. The second dataset consisted of publicly available questions and answers authored by anonymous users on “Naver Jisik-in (Naver Knowledge iN),” a widely used online question and answer (Q&A) platform [29]. Both datasets were publicly accessible and unrestricted. To distinguish between the 2 datasets, the official FAQs provided by the Korean Medical Association and the National Center for Mental Health Portal were referred to as provider-generated content, whereas the questions collected from Naver Knowledge iN were referred to as consumer-generated content. In this study, the term “provider” refers to official institutions that provide health information on ADHD. However, because members of the general public search for and consume health information in online community environments, they are referred to as “consumers” or the “online community.” These terms collectively refer to anyone who accesses information through online communities, including patients, caregivers supporting patients, and members of the general public who suspect they may have a condition.

### Data Collection

All provider-generated ADHD FAQs and their corresponding answers were collected manually, consisting of 88 Q&A pairs. Online community questions and their corresponding responses were collected by accessing the open API of the online community platform using Python (version 3.10.11; Python Software Foundation) after obtaining an API key through registration with the platform provider (ie, Naver).

A 2-step approach was used to identify questions highly relevant to ADHD in the online community. We first retrieved and reviewed the 1000 most recent questions on “ADHD” posted between December 5, 2023, and November 28, 2025. A total of 1000 ADHD-related questions were tokenized, and a word frequency analysis was conducted to identify commonly occurring terms. The terms, listed in descending order of frequency, were as follows: “symptoms,” “treatment,” “child,” “adult,” “attention,” “pediatric,” “concentration,” “diagnosis,” “hospital,” “examination,” “early stage,” and “inquiry.” Among these terms, “attention” was excluded from further analysis because it is inherently embedded in the disease name, ADHD. Similarly, “pediatric” was excluded during the data retrieval process because it semantically overlaps with “child.”

A second round of search and retrieval was conducted using the query format “ADHD AND high-frequency keyword,” with 100 questions extracted per keyword across the 10 identified terms (yielding 1000 questions). After removing duplicate entries and conducting preprocessing, 4988 online community questions and their corresponding answers were retained for final analysis.

### Data Analysis

#### Embedding Models and Similarity Computation

In this study, 3 multilingual sentence transformer–based embedding models were considered: paraphrase-multilingual-MiniLM-L12-v2 (MBERT) [30], Language-Agnostic BERT Sentence Embedding (LaBSE) [31], and multilingual-E5-large-instruct (mE5L) [32]. These models differ in terms of architectural scale and training objectives. MBERT is a lightweight Mini Language Model-based encoder (approximately 100 million parameters) optimized for efficient large-scale sentence similarity computation [30]. LaBSE, a larger dual-encoder model (approximately 470 million parameters), was designed to produce language-agnostic semantic representations across multilingual corpora [31]. In contrast, mE5L (approximately 560 million parameters) was optimized for semantic retrieval and query–text matching through contrastive and instruction-based training, making it particularly suitable for identifying semantically aligned question pairs [32]. All 3 models functioned as representation encoders that generated dense embedding vectors. We did not fine-tune the embedding models for the following 3 reasons. First, extensive pretrained embedding models are used to achieve robust semantic similarity performance on general domain text. This means that fine-tuning primarily refines the activation patterns rather than acquiring new retrieval knowledge. This eliminates the need for resource-intensive domain adaptation [33]. Second, this off-the-shelf approach ensures scalability on standard workstation and methodological reproducibility. This enables providers with limited resources to easily conduct periodic analyses without requiring specialized infrastructure or labeled data [34]. Third, multilingual pretrained encoders, such as MBERT, LaBSE, and mE5L, have demonstrated strong cross-lingual semantic representation capabilities, including Korean, making them appropriate for assessing semantic similarity without additional task-specific training [30-32].

#### Data Preprocessing

The semantic similarity between provider-generated ADHD FAQs and online community questions was calculated at the sentence level. During preprocessing, irrelevant terms were removed using the KoNLPy Korean morphological analysis library [35]. As the provider-generated FAQ dataset was relatively small, a 4988×88 one-to-one similarity matrix was constructed by pairing each online community question with each provider-generated FAQ question. For the response style analysis, each FAQ question was paired with its most semantically similar consumer-generated question from the Q&A dataset, yielding 88 matched question pairs. The corresponding answers to these pairs were then matched to form 88 FAQ–community response pairs, which were analyzed using LLM-assisted content classification.

#### Threshold Optimization Strategy

We optimized the similarity threshold for semantic matching between the 2 datasets by combining distribution- and curvature-based criteria with a performance evaluation based on manual validation. The distribution and scale of similarity scores varied among the embedding models, rendering the application of a uniform absolute threshold across all models inappropriate. Therefore, 3 distinct criteria were used for each model to determine the optimized thresholds. Following manual validation, performance metrics were calculated for each model, and the model that most effectively addressed RQ1 was considered the optimal model. Online community questions with similarity scores exceeding the optimal threshold were considered covered by provider FAQs. The specific steps are as follows.

First, as a distribution-based criterion, the 90th percentile of the similarity score distribution was selected as a candidate threshold to capture a relatively high semantic similarity within the overall distribution of the questions. Percentile-based thresholding methods have been used to identify highly similar regions within the distribution [36]. Prior research on social media text similarity has identified the upper 96th percentile as a criterion for high similarity, and a semantic similarity threshold of 0.7 has been suggested to achieve an optimal balance between relevance and coverage when comparing texts [37]. In this study, the 90th percentile of the similarity score distribution was selected as a simple distribution-based heuristic to retain only the highest similarity pairs while excluding the majority of low-similarity matches. Hereafter, the 90th percentile threshold is referred to as “Q90.”

Second, as a curvature-based approach to threshold selection, the “Kneedle algorithm” was applied to identify an inflection point in the cumulative coverage curve. The Kneedle algorithm detects the “knee point” at which the rate of increase in coverage begins to slow down by examining changes in the shape of the curve [38]. This approach is less sensitive to noise or minor fluctuations in the distribution, making it suitable for identifying meaningful turning points in empirical data [39]. The threshold derived from this curvature-based method was considered a candidate boundary point for subsequent validation and performance comparison. Hereafter, the curvature-based threshold is referred to as “Knee.”

Third, to further refine the threshold optimization beyond the distribution- and curvature-based criteria, a data-driven validation procedure was conducted. Threshold optimization was conducted through manual validation within a 2-stage framework comprising a calibration phase, followed by a confirmation phase [39,40]. Hereafter, this threshold is referred to as “Opt.”

Manual validation was conducted by the first author (JB) and an external reviewer. JB was a doctoral student in nursing informatics with 4 years of experience caring for children with ADHD in community-based residential settings and prior experience in natural language proNatural Language Processing research. The external reviewer was a school nurse with a master’s degree in child and adolescent psychiatric nursing, professional experience supporting students with ADHD, and participation in the development of AI-based digital chatbots.

The dataset comprised 4988 rows, each derived by selecting the most similar question A for each question B in the question A×question B matrix (88×4988). The calibration dataset categorized the entire dataset into 3 primary zones, determined by the thresholds established by the Q90 and Knee analyses conducted previously. These zones comprised a similar zone, which exceeds the Q90 and Knee thresholds; a dissimilar zone, which does not surpass either threshold; or a gray zone, which contains similarity values between the 2 thresholds. Thirty samples were extracted from each zone, resulting in 90 samples per model and 270 samples in the calibration dataset.

Two reviewers independently evaluated whether each question pair conveyed the same contextual intent and assigned binary labels accordingly. Interrater agreement was assessed using Cohen κ coefficient [41]. Discrepancies were resolved through written feedback: the author identified and justified disagreements, which the external reviewer then reviewed and either accepted or rejected to finalize the labels. For each model, the threshold was determined by conducting a grid search based on the final labels during the calibration phase. Possible similarity scores for each model were set as candidate thresholds, and pairs with scores above the threshold were predicted to be similar. Subsequently, the manually validated labels and predictions were compared to calculate the *F*_1_-score, precision, recall, and accuracy. As this study aimed to explore unmet needs based on the semantic similarity between questions, the balanced identification of question pairs with similar intent is more important than simple overall classification accuracy. Accordingly, the *F*_1_-score, which is the harmonic mean of precision and recall, was the primary metric for selecting the threshold of each model. During the confirmation phase, the *F*_2_-score was reviewed to support the exploration phase in identifying unmet needs for the final automated model selection. To determine an adequate validation sample size, the observed *F*_1_-score from the calibration dataset was used to estimate the minimum number of samples required for the confirmation phase. The required sample size was calculated to achieve a 2-sided 95% CI with a target width of 0.20 [42].

#### LLM-Assisted Topic Modeling for Identifying Consumer Information Needs

To identify unmet information needs reflected in the extracted sentences, we applied the LimTopic approach [43], an LLM-assisted hybrid topic modeling framework. The LimTopic approach involves a series of analyses, such as sentence embedding using a BERT-based model, topic modeling, and topic labeling and summarization using LLMs. Traditional topic modeling methods, such as Latent Dirichlet Allocation [44], often have limited interpretability of the topics captured. Although BERTopic [43] improves interpretability by clustering semantically similar sentences into coherent topic groups, generating meaningful topic labels remains challenging. LimTopic addresses these limitations by incorporating LLM-based summarization to produce topic titles and summaries.

Within the LimTopic framework, the paraphrase-multilingual-MiniLM-L12-v2 model was used for sentence embedding because it was the only multilingual model among the tested sentence transformers that supported multilingual text processing [43]. As reported in the LimTopic framework, the BERTopic (Maarten Grootendorst) + GPT-4 Turbo (OpenAI) few-shot configuration achieved the best performance in terms of Silhouette and Coherence scores [43]. Few-shot prompting with GPT-4o-mini (OpenAI) was applied for topic labeling and summarization because the GPT-4 Turbo API service was no longer available[45].

#### LLM-Assisted Content Analysis

To propose consumer-oriented information provision strategies, LLM-assisted deductive content analysis examined both the information providers and the community’s answers to questions and classified their overall response characteristics. Recent studies have shown that LLMs can successfully support qualitative coding and thematic summarization when guided by structured prompts and predefined coding frameworks [46,47]. In this study, an LLM was used as a coding assistant. The codebook for content analysis was based on the existing Centers for Disease Control and Prevention Clear Communication Index [48,49]. There are 7 main index principles, of which 4—communicative function, evidence and authority signaling, outcome framing, and actionability—were selected as dimensions for content analysis. The categories and operational definitions were formulated based on previous research (Table 1). The concept of communication function refers to the role of the information provided by the provider. Evidence and authority signaling determine how the rationale for a response is presented [50,51]. Outcome framing examines how benefits, risks, and outcomes are communicated within the response content [52,53]. Actionability assesses the extent to which a response offers guidance on potential consumer actions [54]. These definitions were directly integrated into prompting instructions.

**Table 1.** Code book for large language model–assisted content analysis.

Dimension and category	Operational definition
Communicative function	
Informational guidance	Its main function is to provide factual, conceptual, causal, options-related, procedural, and actionable information.
Emotional support	Providing emotional support, such as validation of feelings, comfort, reassurance, and encouragement, is the primary function.
Experiential context	Explanation centered on actual experiences or case observations of individuals, patients, caregivers, and professionals.
Mixed or other	No category is clearly predominant.
Evidence and authority signaling	
Research evidence only	Research, scientific literature, clinical practice guidelines, public health agencies, and explicitly citing research findings as evidence.
Professional authority only	Presented based on expert opinion, clinical experience, professional authority, and expert consensus without research evidence.
Both	There are both research evidence signals and expert authority signals.
Neither	No signal from either.
Outcome framing	
Gain or benefit focused	Primarily emphasizes the benefits, improvements, positive outcomes, and risk reduction gained from engaging in the recommended behavior.
Risk or loss focused	Primarily emphasizes losses, harm, deterioration, and complications associated with inaction or continued risky behavior.
Balanced	Present both benefits and risks or losses in a relatively balanced manner.
No explicit outcome information	Does not explicitly emphasize the consequences of actions or choices.
Actionability	
Specific actionable recommendation	The response provides at least one concrete action that the consumer can take and includes specific directions about how, when, under what conditions, or in what sequence to perform the action.
General behavioral recommendation	The response recommends health-related behavior but provides limited or no details about how, when, or under what conditions it should be performed.
Referral or help-seeking recommendation only	The response only recommends consulting, contacting, or visiting a health care professional, pharmacist, clinic, emergency service, or other support sources without providing another consumer-performable action.
No behavioral recommendation	The response does not provide any action that the consumer can take; it only explains, describes, evaluates, shares experiences, or provides emotional support.

The model used for the analysis was GPT-4o-mini. Prior evaluations indicate that GPT-4o-mini achieves its best performance under relatively simple few-shot prompting, with no substantial improvement from more complex reasoning prompts [55]. Considering its cost efficiency and reliable performance on structured classification tasks, GPT-4o-mini was selected for analysis [55,56]. For each response, the LLM was instructed to assign one category per dimension according to the operational definitions in the codebook. The output followed a structured response format. The classifications produced by the model were retained, and interpretive considerations were addressed in the *Discussion* section. All outputs were subsequently reviewed by the authors to ensure their interpretive validity [57].

### Ethical Considerations

This study was reviewed and approved by the Institutional Review Board of Seoul National University (IRB E2508/002-008).

## Results

### Overview

This section sequentially addresses the 3 RQs. First, RQ1 was examined by analyzing the threshold optimization for semantic similarity and the subsequent coverage assessment of online community questions by provider-generated FAQs. Next, RQ2 was examined by identifying and characterizing online community questions that remained uncovered by FAQs, highlighting unmet information needs. Finally, RQ3 was explored by comparing response styles between provider-generated FAQ answers and online community responses using structured LLM-assisted content analysis.

### Threshold Optimization for Semantic Similarity

**Figure 1.**
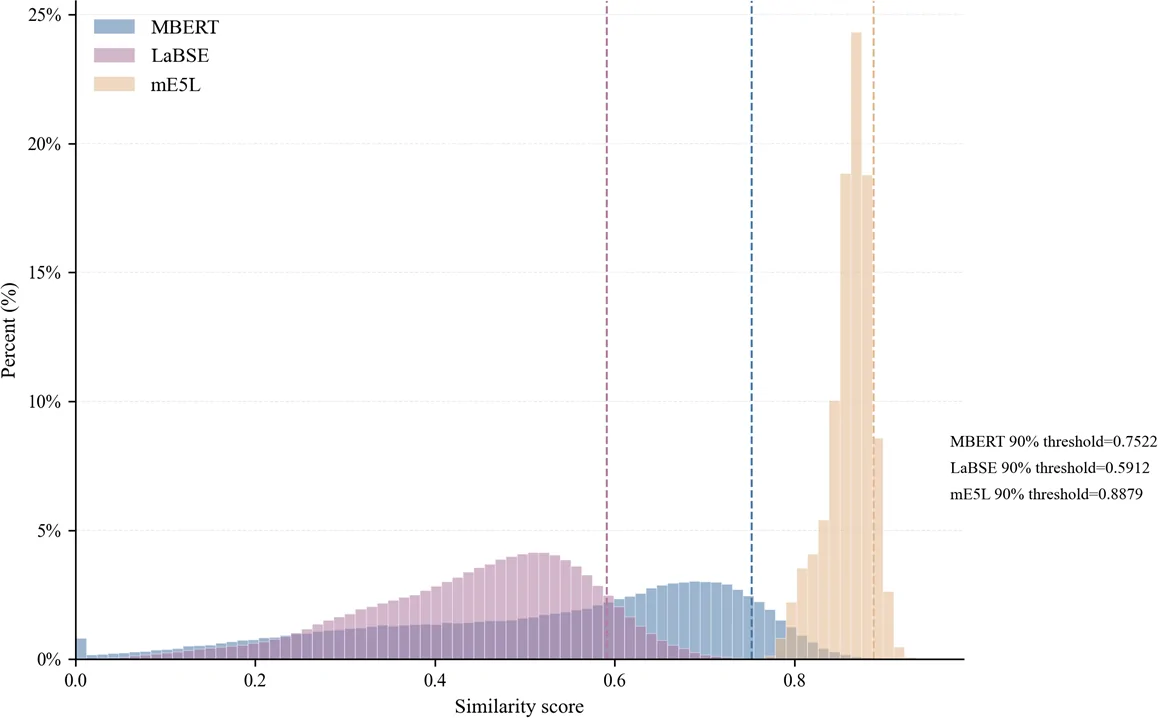
Distribution-based similarity threshold. The figure shows the pairwise similarity scores across all provider frequently asked questions (FAQ)–online community question pairs for each embedding model. Dashed lines indicate model-specific 90th percentile thresholds ( paraphrase-multilingual-MiniLM-L12-v2 [MBERT]=0.7522, Language-Agnostic BERT Sentence Embedding [LaBSE]=0.5912, and multilingual-E5-Large-instruct [mE5L]=0.8879). By limiting matches to the top 10% of similarity scores, these thresholds provided stringent criteria for determining FAQ coverage.

For each word embedding model, a similarity matrix with dimensions 4988×88 was constructed to calculate the pairwise similarity between provider-generated FAQs and online community questions. The initial distribution-based criterion was the 90th percentile of the similarity score distribution. The resulting threshold values were 0.7522 for MBERT, 0.5912 for LaBSE, and 0.8879 for mE5L (Figure 1).

**Figure 2.**
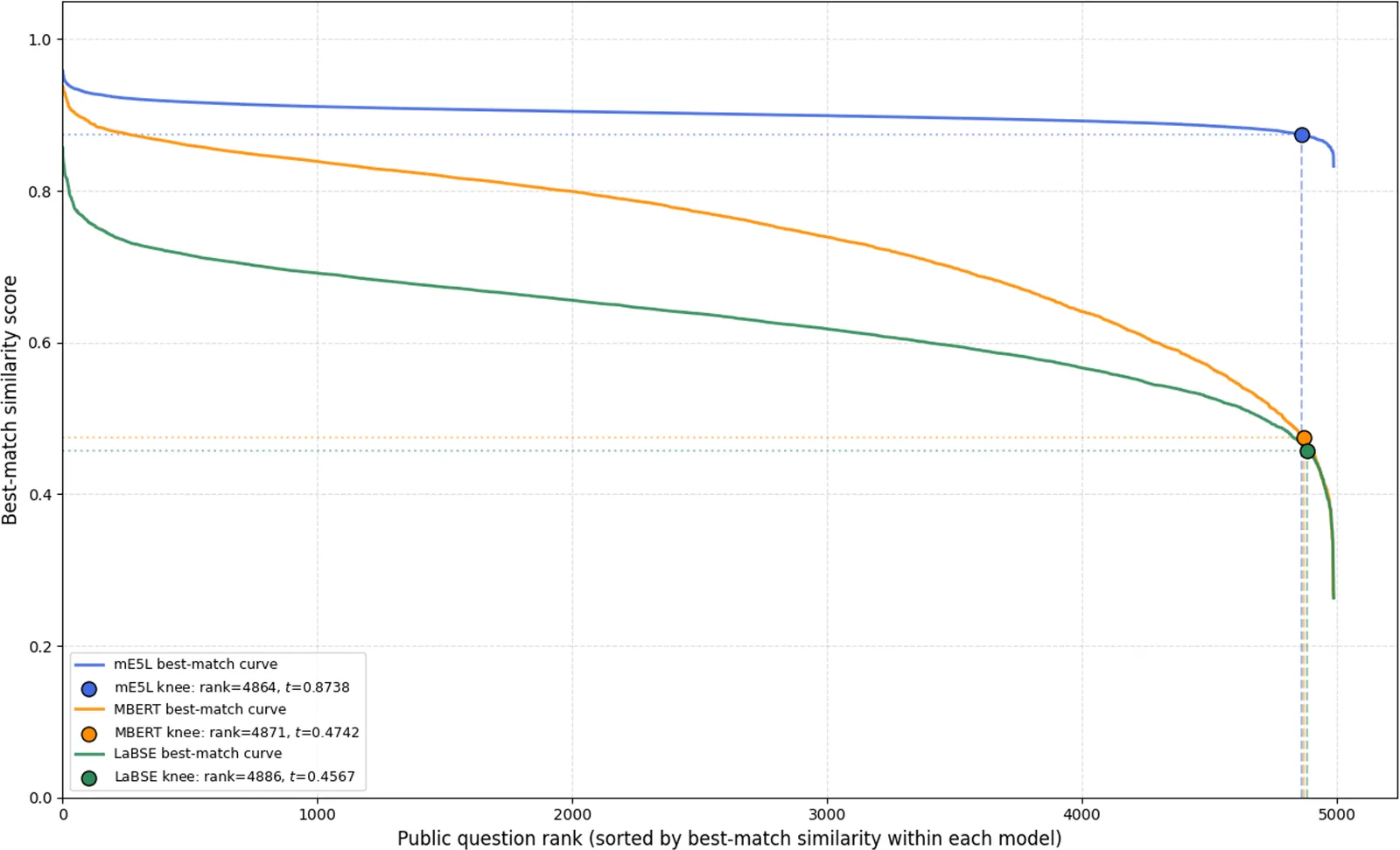
Curvature-based threshold identification using the Kneedle algorithm. For each embedding model, the 4988 online community questions were ranked based on their highest similarity score across all provider frequently asked questions (FAQs). The Kneedle algorithm identified the point at which the best-match similarity curve declined sharply, and the corresponding similarity score was selected as the model-specific threshold (*t*). This knee point represents the point of diminishing coverage gains, beyond which additional FAQ matches yield little further increase in the question coverage. LaBSE: Language-Agnostic BERT Sentence Embedding; MBERT: paraphrase-multilingual-MiniLM-L12-v2; mE5L: multilingual-E5-Large-instruct.

As the second threshold criterion, cumulative distribution curves were constructed by listing questions similar to question B to determine the final similarity values across a range of questions. The knee point, where the rate of change became notably more gradual, was identified from these curves and used as a threshold. This knee point was not determined by a simple slope measurement but corresponded to the point at which the coverage performance reached its maximum value. Thus, this threshold indicates the point beyond which further increases in similarity do not significantly enhance the FAQ’s coverage of online community questions. The Kneedle algorithm, implemented using the Python Kneed library, was used to identify the knee points in the cumulative similarity distributions [38]. The Kneedle algorithm allows the adjustment of inflection point detection by setting the sensitivity, which was set to the default value of 1. The resulting knee point thresholds were 0.4742 for MBERT, 0.4567 for LaBSE, and 0.8738 for mE5L (Figure 2).

As the third threshold criterion, the threshold optimization step was validated manually. Optimization was performed in 2 phases (calibration and confirmation), and the results are presented in Tables 2 and 3. The Opt threshold consistently yielded higher *F*_1_-scores than the heuristic threshold selection methods across the evaluated embedding models. Under the Opt threshold, MBERT achieved the best overall balance between recall and precision, resulting in the highest *F*_1_-score. Therefore, the optimized threshold identified using the validation dataset was adopted as the reference threshold for subsequent automated semantic similarity classification.

**Table 2.** Comparison of similarity threshold selection methods across word embedding models calibration phase.

Model and method	Threshold	*F*_1_-score	Recall	Precision	Accuracy
mE5L^a^					
Q90^b^	0.8879	0.2041	0.2632	0.1667	0.5667
Knee^c^	0.8738	0.3797	0.7895	0.2500	0.4556
Opt^d^	0.8751	0.3896	0.7895	0.2586	0.4778
MBERT^e^					
Q90	0.7522	0.4815	0.5417	0.4333	0.6889
Knee	0.4742	0.5000	0.8750	0.3500	0.5333
Opt	0.5981	0.5753	0.8750	0.4286	0.6556
LaBSE^f^					
Q90	0.5912	0.2632	0.6250	0.1667	0.6889
Knee	0.4567	0.2353	1.0000	0.1333	0.4222
Opt	0.6471	0.5000	0.3750	0.7500	0.9333

a mE5L: multilingual-E5-Large-instruct.

b Q90: similarity threshold based on the 90th percentile of the similarity score distribution.

c Knee: similarity threshold identified at the knee point of the cumulative similarity distribution using the Kneedle algorithm.

d Opt: optimized similarity threshold derived through validation-based performance evaluation using manually labeled question pairs.

e MBERT: paraphrase-multilingual-MiniLM-L12-v2.

f LaBSE: Language-Agnostic BERT Sentence Embedding.

**Table 3.** Comparison of similarity threshold selection methods across word embedding models confirmation phase.

Model and method	Threshold	*F*_1_-score	*F*_2_-score	Recall	Precision	Accuracy
mE5L^a^						
Q90^b^	0.8879	0.2917	0.3431	0.3889	0.2333	0.6222
Knee^c^	0.8738	0.3846	0.5682	0.8333	0.2500	0.4667
Opt^d^	0.8776	0.4242	0.5833	0.7778	0.2917	0.5778
MBERT^e^						
Q90	0.7522	0.3830	0.4592	0.5294	0.3000	0.6778
Knee	0.4742	0.3896	0.5859	0.8824	0.2500	0.4778
Opt	0.7660	0.4500	0.4945	0.5294	0.3913	0.7556
LaBSE^f^						
Q90	0.5912	0.2326	0.3049	0.3846	0.1667	0.6333
Knee	0.4567	0.2466	0.4018	0.6923	0.1500	0.3889
Opt	0.5221	0.3051	0.4592	0.6923	0.1957	0.5444

a mE5L: multilingual-E5-Large-instruct.

b Q90: similarity threshold based on the 90th percentile of the similarity score distribution.

c Knee: similarity threshold identified at the knee point of the cumulative similarity distribution using the Kneedle algorithm.

d Opt: optimized similarity threshold derived through validation-based performance evaluation using manually labeled question pairs.

e MBERT: paraphrase-multilingual-MiniLM-L12-v2.

f LaBSE: Language-Agnostic BERT Sentence Embedding.

For the calibration phase, the calibration validation set consisted of 90 question pairs for each of the 3 embedding models (mE5L, MBERT, and LaBSE), totaling 270 pairs. The Cohen κ coefficient for interrater reliability between the 2 independent reviewers was 0.8596. Among the 270 reviews, 11 discrepancies were observed. The author subsequently provided comments and feedback to the external reviewer, leading to a consensus. The external reviewer accepted 6 of the 11 discrepancies, with the author agreeing with the external reviewer’s assessments. After the calibration validation, the performance metrics for each model are presented in Table 2. In the information retrieval evaluation literature, in situations where relevant items are sparse or exploratory searches are required, precision and recall are considered key metrics rather than accuracy, and the *F*-measure, which reflects the balance between these 2 indicators, is widely used. Accordingly, this study set the *F*_1_-score as the primary metric for model and threshold selection. The final embedding model was selected by calculating the sample size for the additional review dataset based on the *F*_1_-score identified during the calibration validation process [42]. The 95% CI for the *F*_1_-score was constrained to a width of 0.20 or less. Applying this criterion to the provided code, the minimum required sample size was determined as 246. Consequently, 270 samples were selected for the confirmation set using the same method as in the calibration phase. Subsequently, to finalize the model’s threshold, each reviewer independently conducted and adjusted a manual review of 270 questions. Cohen κ coefficient for interrater agreement was 0.8282. In the second validation, there were 14 differing opinions out of 270, which were conveyed to the external reviewer in the same manner as in the first validation. The external reviewer accepted 10 of the 14, and the researcher also agreed; therefore, the final decision was made. The interrater agreements are presented in Multimedia Appendix 1. After confirmatory validation, the performance metrics for each model are presented in Table 3. By comparing the threshold criteria for each model on the test validation set, the optimal performance in terms of the *F*_1_-score was observed with an MBERT manual grid search threshold of 0.7660 (*F*_1_-score=0.4500). Under these conditions, the precision was 0.3913, and the accuracy was 0.7556, both of which were the highest, and the overall balance of performance was also the best. In contrast, under the *F*_2_-score criterion, the Kneedle threshold of MBERT yielded the highest value (*F*_2_-score=0.5859; recall=0.8824). This suggests that adopting a lower threshold may be advantageous in the exploration of unmet needs. However, as the precision was lower and the risk of false positives increased, the *F*_1_-score was used as the primary metric in the final model selection, with the *F*_2_-score and recall considered auxiliary indicators.

### Coverage of Online Community Questions by FAQs

**Figure 3.**
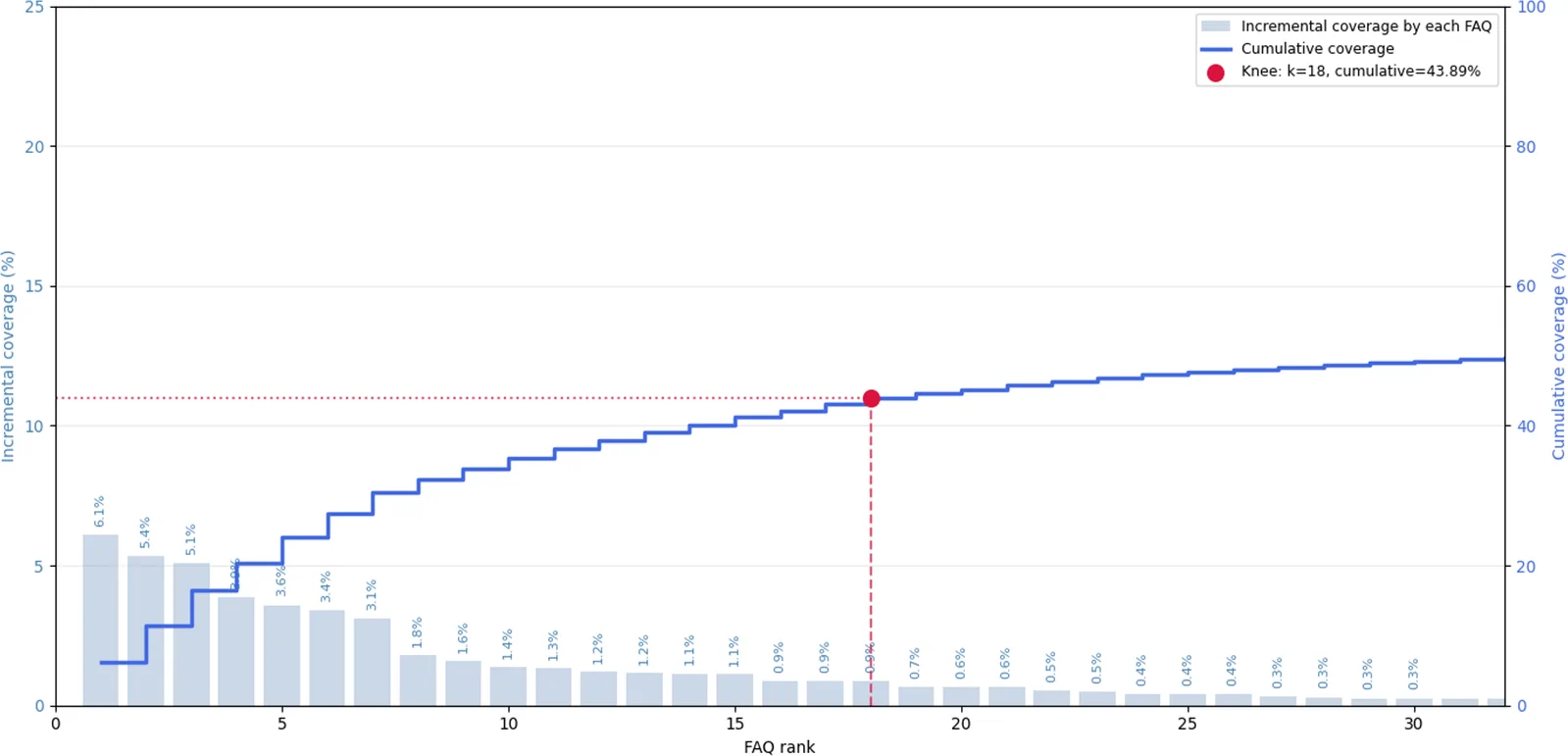
Cumulative coverage of online community questions by ranks using the paraphrase-multilingual-MiniLM-L12-v2 (MBERT) model provider. Frequently asked questions (FAQs) were ranked according to the proportion of online community questions covered using an optimal MBERT similarity threshold. The bars represent the incremental coverage contributed by each FAQ, and the blue line represents the cumulative coverage. The knee point (FAQ rank=18) indicates the point of diminishing coverage gains, beyond which additional FAQs contribute only marginal gains in overall question coverage.

When the similarity score, calculated from the closest semantic match among the FAQs for a consumer’s question, is above 0.766, it indicates that the health information provider understands the consumer’s intent. The author expressed this as FAQ covering questions from the online community. With the best similarity threshold for MBERT set at 0.766, 2598 of the 4988 online community questions were matched to at least one FAQ item, achieving a coverage rate of 52.09%. To examine how individual FAQs contributed to this coverage, the FAQs were ranked according to the number of online community questions that matched each item. As a single FAQ could be matched with multiple online community questions, the ranking reflected the relative contribution of each FAQ to overall coverage. Cumulative coverage across the ranked FAQs was analyzed using the Kneedle algorithm [38]. The knee point was determined at rank 18, signifying that FAQs constituted approximately 43.89% of the matched online community questions (Figure 3).

Table 4 presents a list of community questions that most closely align with the top 10 FAQs, as depicted in the cumulative distribution curve above. The FAQs were ranked according to the number of community questions that received judgments surpassing the threshold of 0.766.

**Table 4.** Top-ranked frequently asked questions (FAQs) covering consumer questions.

FAQ rank	FAQ	Count (n)	Best matched community question	Best similarity
1	Are only those who were diagnosed with ADHD^a^ in childhood considered to have adult ADHD?	305	ADHD is known to develop at a young age, but this year, at twenty-one years old, I was diagnosed with it. Was the diagnosis made incorrectly, or is it really possible to have it? Is it something that doesn’t go away naturally? Please let me know!	0.924
2	How should I come to terms with my child’s ADHD?	267	It seems that my child has ADHD. I’m having trouble coping—what can I do to help them improve?	0.906
3	Is adult ADHD treatable?	253	I have been diagnosed with adult ADHD and would like to receive treatment. Could you tell me what treatment options are available?	0.921
4	Should ADHD be treated even though my child is still young?	193	The child’s temperament test results were as follows: I am wondering whether it would be good to add an ADHD assessment.	0.928
5	Impulsive shopping could mean you have ADHD	179	As an adult with ADHD, I keep forgetting things and getting called out for it, and I also get scolded frequently at work. I am wondering whether I might have adult ADHD.	0.897
6	Should I tell my child about their ADHD diagnosis?	170	The child displayed a lot of impulsive behaviors, but they were not at a level that could be dismissed as just being young. I am wondering whether it might be ADHD and would like to know what the diagnostic criteria for ADHD are. In addition, I would like to know more details about the treatment methods.	0.916
7	School Life and Teachers of Children with ADHD	154	Children with ADHD who enter elementary school experience pediatric ADHD. They are said to wander around restlessly and disrupt classes. Although I want to pursue treatment, I feel lost regarding the best approach. Please let me know the available treatment options.	0.868
8	If a parent has ADHD, is their child also more likely to, have it?	90	I have heard that ADHD can be hereditary. I am curious about the actual probability of its occurrence when there is family history.	0.908
9	What are the available treatment options for adult ADHD?	80	What are the treatment options for adult ADHD? I am curious if there are any treatments other than medication. Please provide a detailed answer.	0.938
10	How often do childhood and adolescent ADHD persist in adulthood?	69	The ADHD symptoms in my child with ADHD are being managed to some extent with medication and therapy. However, I wonder whether these symptoms naturally disappear over time or if they often persist in adulthood. I would also like to know if there are any characteristics that make it easier for children to improve their performance.	0.901

a ADHD: attention-deficit/hyperactivity disorder.

To determine whether it is meaningful to assume that FAQ questions with a similarity score above a certain threshold cover consumers’ information needs, the author specifically examined the responses corresponding to the 18 FAQs up to the knee point. The author examined whether these answers were appropriate responses to the intent reflected in the questions most similar to the 18 FAQs. Of the 18 answers, 16 (88.9%) were deemed to adequately address the consumers’ inquiries, while 2 (11.1%) were deemed insufficient. One question appeared likely to be answered by describing the drug’s effects, while the intent of the other was misjudged. Therefore, we concluded that comparing question similarity was a feasible and practical approach for quickly identifying consumers’ unmet information needs.

### Online Community Questions Not Covered by FAQs

According to the similarity comparison analysis, the proportion of questions in online communities not covered by the FAQs was 47.91%, totaling 2390 questions. By analyzing these, we identified the information needs of consumers that were not addressed in the FAQ. To explore the unmet information reflected in these questions, the researchers conducted an analysis using the LimTopic framework [43]. LimTopic used UMAP and BERTopic, followed by GPT-4o-mini. The parameters for these methods are accessible via publicly available codes. LimTopic identified 118 topics, although numerous instances of duplicate content were observed. To elucidate unmet needs, the researcher initially categorized the data and sought a review from the same external expert. Subsequently, the researcher integrated the feedback to finalize the study results. The final unmet need outcomes are presented in Table 5.

**Table 5.** Unmet attention-deficit/hyperactivity disorder (ADHD) information needs of consumers.

Category	Core unmet needs	Representative questions
Challenges in locating hospitals capable of providing a diagnosis and facilitating access to medical care	Lack of information on hospitals capable of testing within the region Lack of transparency in retesting and represcription procedures during referral/transfer	“I was receiving psychiatric treatment for ADHD, including medication, after being diagnosed at a psychiatry clinic, but I have to move now. If I want to continue treatment at a hospital in the new area, do I need to undergo ADHD testing and treatment again?” “I was tested at a psychological counseling center due to suspected ADHD and was advised to visit a hospital. Are there any psychiatric hospitals in Seoul where I can get an appointment immediately?”
Uncertainty regarding the reliability of diagnostic tests and procedures	The disparity between self-diagnosis and professional diagnosis Coupled with a lack of trust in the economic burden associated with testing methods, the high cost of tests Uncertainty surrounding adult ADHD self-diagnosis	“I am considering the possibility of adult ADHD and, while searching for relevant information, I want to get a definitive diagnosis at a hospital. How is diagnosis conducted in hospitals?” “I am considering getting tested for ADHD, but I have heard that it can be expensive. In addition, what types of tests were conducted? Are the test results reliable? Is there credibility to the tests? There are many self-diagnosis tests for ADHD available on the Internet. Can these be used to determine whether someone has ADHD?”
Burden of examination and treatment costs	The economic burden of high-cost tests (Wechsler, EEG^a^, etc)	“I am curious if there are general cost differences between university hospitals and private psychiatric clinics, as well as how the portions covered by health insurance differ from the out-of-pocket, non-insured expenses. If you could let me know the approximate average cost range, it would be helpful for my planning.”
Disadvantage due to medical history	Lack of information on disadvantages of diagnosis history for private health insurance enrollment and claims, concerns Restrictions on employment and social activities due to medical history	“I am a 21-year-old woman, and I suspect that I may have adult ADHD. Therefore, I am considering going to the hospital to get a diagnosis. If I receive a diagnosis and start taking medication, how will it affect my insurance? I currently have an indemnity insurance policy that I signed up for as a child, and it will mature when I turn 30. However, I am worried that my premiums might increase significantly when I renew them.” “I think I might have adult ADHD, and I am considering treatment. Will this put me at a disadvantage when searching for a job?”
Anxiety about the efficacy and side effects of pharmacotherapy	Delayed effects of stimulants such as Concerta and Medikinet Lack of information on coping with side effects (insomnia, loss of appetite, brain fog, etc) Insufficient information on treatment adherence (dosage, intervals, etc)	“I have been taking antidepressants, bipolar medication, and Medikinet, and then switched to Concerta, but nothing seems to have changed. I cannot tell if it is depression or emptiness, but these anxious feelings have continued for about four months even while taking medication, and now it is just getting too hard. Does this mean that the medication does not suit me?” “At first, when I started taking Medikinet, I did not procrastinate and thought it was effective, but over time, it seemed like the medication was not working, and I was not sure about Concerta either. After 3‐4 h, the depression became extremely severe. Not only do I feel like crying, but I also experience lethargy, extremely negative thoughts, persistent fatigue and sleep much more.” “The university hospital professor recommended taking the medication for about 3 to 4 years and said there is about a 60% chance of a cure, so after much deliberation, we finally decided to start the medication, but I am worried because there are side effects such as loss of appetite and sleep problems...”
Information needs regarding nonpharmacological alternative therapies	Lack of reliable information on nonpharmacological alternatives such as Korean medicine, nutritional supplements, dietary interventions, speech therapy, and social skills training	“I would like to know whether ADHD can be treated with Korean medicine and how effective it is. Can acupuncture or herbal medicine improve a child’s concentration? I am also curious about the typical duration of the treatment. I am so worried about my child’s future that I can hardly sleep at night. It would be a great relief if there were a way to help without medication.”
Inadequate integrated management of comorbidities (depression, tic disorders, autism, etc)	Lack of systems for comorbidity (secondary, multiple) differentiation and concurrent treatment	“If someone has both depression and ADHD, is depression treated first, followed by ADHD treatment? When I take ADHD medications, the noisy thoughts in my head disappear, and I can focus on work, which is good, but the more I use them, the more the dosage increases, and as a result, I experience a vicious cycle where symptoms of other conditions, such as panic disorder, worsen.”
Lack of information and caregiving support for parents of children with ADHD	Coping strategies for behavioral problems in children and adolescents, insufficient information on hospital selection, inadequate parental stress support system Stigma and guilt experienced by parents of children with ADHD	“ADHD child... The medication does not seem to work, and we cannot send them to a special education class. This situation is exhausting. What are the alternatives in this case?” “I regret so much that, instead of focusing on my child’s many strengths, I let a few shortcomings make me anxious and distressed and end up giving those side effect-ridden medications. Even now, if I stop the medication and change my parenting approach, could things get better? I wish I could turn back time. Please advise on the steps I should take.”
Late-onset awareness in adulthood and social stigma and relationship conflict	Identity confusion after adult diagnosis Lack of understanding in family and workplace Insufficient resources to cope with social stigma	“I used to think I got along well with others because I was generally energetic and talkative, but as I got older, I increasingly found myself misunderstood, or my relationships became awkward due to my way of speaking and communication style, which became a source of concern.Since childhood, I was often scolded by teachers for being unable to concentrate during class, and I think I may have had ADHD symptoms since then. Now that I have realized the symptoms this late and am receiving treatment, is it still possible to improve?”
Lack of practical coping resources for decline in activities of daily living	Lack of medical and social resources for improving daily functioning such as concentration, time management, emotional regulation, sleep, and prevention of losing items	“It takes me a long time to understand things and get used to things. I did not realize this when I was in school, but I have noticed it often since I started working. That is why I think I spend at least 30 minutes to 1 h more each day on job proficiency than others. What is the best way to handle this?”
Information needs for supporting school life and academic performance	Support for learning and academic performance Adaptation to school and classroom life Social relationships within the school	“What is the relationship between ADHD and learning disabilities? I am wondering whether ADHD can cause learning disabilities. If both conditions are present, what kind of treatment or support is needed? Is it possible to treat concentration issues such as ADHD at a Korean medicine clinic? I am curious about which department to visit and whether acupuncture or herbal medicine treatments can help improve concentration.” “I want to find natural ways to improve my concentration without medication. Are there any good methods for enhancing focus while studying?” “I often hear things like “You’d be normal if you just kept your mouth shut,” “You seem disabled sometimes,” and “Can you just sit still” from friends around me. I have trouble controlling my anger, and when I get angry, I yell loudly, get extremely agitated, and have even thrown things. Would medication help?”
Information needs regarding independent diagnostic testing for adolescents	Diagnosis of ADHD without parental consent Solutions for conflicts with parents who do not wish for an ADHD diagnosis	“I would like to go with my parents, but I worry they might think I am making too much of a fuss, so I just want to go alone. Would that be possible?” “I am a high school student, and I want to get tested for ADHD. I heard that my parents need to accompany me for the test, so I want to tell them, but I’m afraid they’ll scold me or ignore me, so I’m scared to bring it up.”

a EEG: electroencephalography.

### Differences Between FAQ Responses and Online Community Responses

**Figure 4.**
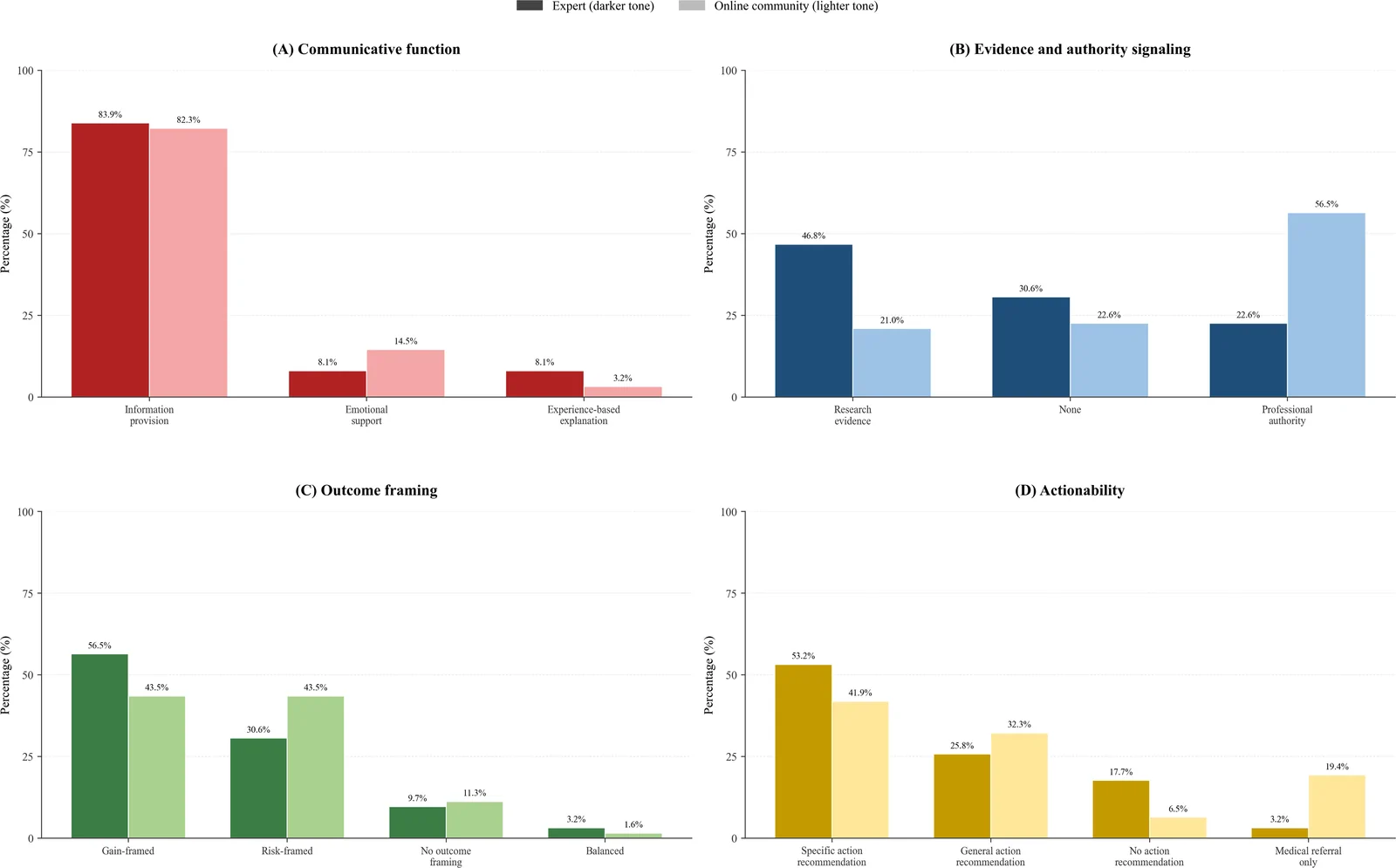
Large language model–assisted content analysis: a comparison between information provider and online community answer styles. Percentages of responses across the 4 content dimensions were compared between provider frequently asked questions’ answers and online community answers: (A) communicative function, (B) evidence and authority signaling, (C) outcome framing, and (D) actionability.

We conducted a structured LLM-assisted content analysis to compare expert-generated FAQ answers with online community responses. The purpose of this analysis was not to evaluate the correctness of the responses but to explore the differences in the stylistic and communicative characteristics of responses. To this end, among the 88 FAQs, the answers to 62 questions for which the highest similarity with any consumer question exceeded the similarity threshold, along with the corresponding answers to the online community questions, were designated as analytical data. A predefined codebook (Table 1) was used for the analysis. Figure 4 shows the distribution of response classifications across the 4 dimensions.

In this study, chi-square tests were used to explore differences in the distribution of questions and responses across 4 dimensions (communicative function, evidence and authority signaling, outcome framing, and actionability), between provider-generated FAQs and online community responses. The chi-square statistics and *P* values for each area helped determine whether there were significant differences between the 2 groups. First, in the communicative function dimension (Figure 4A), the results showed no significant difference (*χ*²_2_=2.44; *P*=.30) between the 2 groups. This means that both FAQs and community responses showed similar patterns in providing information, emotional support, and experience sharing. Second, the evidence and authority signaling dimension (Figure 4B) showed a significant difference (*χ*²_2_=15.85; *P*<.001). This means that expert-generated FAQs present research evidence and professional authority more clearly, whereas community responses often include fewer expert signals and more personal opinions than the FAQs. Third, for the outcome framing dimension (Figure 4C), the chi-square test showed no significant difference (*χ*²_3_=2.83; *P*=.42). Both groups had similar distributions of gain-, risk-, or balance-focused framing outcomes. Finally, the behavioral actionability dimension (Figure 4D) showed a significant difference (*χ*²_3_=11.68; *P*<.001).

## Discussion

### Principal Findings

This study examined the extent to which existing provider FAQ on ADHD content addressed the information needs reflected in consumer-generated questions collected from an online community. The findings revealed meaningful gaps between the questions consumers asked and the information provided in the provider FAQ, indicating that some information needs remained insufficiently addressed. In addition, this study demonstrated the feasibility of using an embedding-based approach to systematically assess the extent to which existing FAQ content addresses consumers’ information needs. By combining automated semantic matching with manually validated, model-specific thresholds, the proposed approach offers a scalable means of identifying potentially unmet information needs in large-text datasets.

The provider-generated ADHD FAQ addressed only 52.09% of questions from online communities, leaving 47.91% of consumer needs unmet. Most online community questions were related to only 18 of the 88 FAQ items, with the remaining 70 contributing minimally to overall coverage. These findings suggest that although FAQs are intended for consumers, they only partially meet consumer needs. Therefore, rather than maintaining a large number of FAQs based on existing materials, it is advisable to adopt an information provision strategy that improves FAQ relevance by incorporating topics aligned with consumer needs through continuous monitoring. The proposed directions for information restructuring are as follows.

Health information on ADHD should transition from a disease-centric perspective to one that more effectively supports real-life decision-making. Table 3 outlines fundamental, standardized topics such as diagnosis, treatment, prognosis, parental roles, school adaptation, and genetics. Conversely, Table 5 reveals consumers’ unmet informational needs, which encompass more practical and comprehensive issues. These include challenges in accessing diagnostic institutions, uncertainty about diagnostic reliability, financial burden of testing and treatment, concerns about disadvantages stemming from diagnostic history, anxiety regarding side effects, insufficient information on nonpharmacological alternative therapies, and management of comorbidities. Consumers in online communities expressed a desire for systematic information on access to care and associated costs, including ADHD diagnostic pathways, available medical institutions, estimated expenses, insurance coverage, and financial support systems.

Furthermore, practical strategies should be incorporated to assist consumers in applying medical knowledge for effective self-management. Given that children are the main group affected by ADHD, expanding the focus to encompass details about assistance from educational institutions and the community is crucial. Providing information to ensure adolescents’ access to treatment is essential. We should address the demand for information on whether adolescents can independently access diagnosis and treatment due to social stigma and a lack of parental understanding. Additionally, information on social support systems is necessary. For adolescents facing challenges such as parental opposition to treatment or lack of symptom recognition, accessible counseling services should be offered, along with age-appropriate procedures and support. This highlights the necessity of providing information capable of addressing intricate and context-dependent inquiries about issues such as diagnostic delays, academic assistance, and reductions in daily capabilities, among other matters. Furthermore, concerning adult ADHD, there is a requirement for information on employment limitations due to diagnosis, management of social maladjustment, and treatment strategies for late diagnosis.

Given the concerns about drug side effects, which are seen as a barrier to treatment, and the growing interest in nonpharmacological interventions, such as traditional Korean medicine, play therapy, and social skills training, it is crucial for health information to thoroughly cover the effectiveness, evidence, and appropriate use of these treatments. Referring to the actionability domain results in Figure 4, if FAQ responses—already known for providing actionable advice from official sources—could further develop the expertise of relevant organizations and broaden the information to include formal processes and reliable social support services, it is anticipated that consumers would receive practical information that aligns with their needs.

Automation techniques are necessary to rapidly identify consumers’ health information needs and provide customized information. However, this suggests that approaches involving expert validation are essential. The 3 embedding models used in this study (MBERT, LaBSE, and mE5L) each display different similarity distributions and characteristics, and manual validation confirmed that it is inappropriate to apply the same absolute similarity threshold across models. When 2 experts reviewed the similarity determinations produced by the embedding models, they found that clear judgments could not be made in the following cases: when there are overlapping words within sentences but the intent of the question is not clearly captured (eg, when the same symptom is described but the distinction between pediatric and adult cases differs); when it is the same case but the subject seeking the answer is different (eg, answers differ depending on whether the parent or the child is asking about the same issue); when the key point is simple yet accompanied by excessive explanation; or when names of specific times or unique locations are included. In such cases, it was difficult to rely solely on the results of similarity analysis. Therefore, to rapidly analyze consumers’ unmet needs embedded in large datasets, selecting the optimal similarity threshold through manual validation based on the results of the embedding model’s similarity analysis is an important process in the pipeline for analyzing consumer information needs.

### Comparison With Prior Work

The findings of this study emphasize the existence of various informational gaps at the social and psychological levels, including systemic factors related to ADHD diagnosis and treatment, social stigma, and the disadvantages associated with being diagnosed. These findings align with the need to address stigma and unmet needs [14], correct misconceptions for timely diagnosis and treatment [15], and provide accessible and empathetic online information [16]. The experiences of individuals describing delayed diagnosis due to social stigma, parental conflicts, and social disengagement support previous conclusions [58] that educating people to dispel misconceptions about ADHD can reduce psychological distress and encourage timely diagnosis and treatment. When individuals elaborate on their situations in their inquiries or use phrases such as “I'm struggling,” “I’m so upset. It’s because I raised it wrong,” or “I'm not the only one like this, right?,” it suggests a need for information that provides emotional support alongside factual details. For those expressing such sentiments, responses from the online community group that acknowledge the emotional state of the questioner and show empathy, such as “You must be very worried about your child’s symptoms,” “I hope this answer helps ease your concerns,” and “I fully understand the difficulties you are facing as a parent,” can enhance the effectiveness of the information provided. Furthermore, it was identified that there is a necessity to include details about the diagnostic process, treatment protocol, and multidisciplinary management of common comorbid conditions such as depression and anxiety that frequently occur with ADHD [59,60]. In particular, in cases where individuals have negative views of pharmacological treatment, it is crucial to offer suitable information about ADHD treatments along with explanations of situations where medication is necessary.

Additionally, prior work has verified that consumers generally trust health care professionals because of their roles as physicians, and physicians use this trust to spread health information [61,62]. In light of this, we recognized the importance of being cautious about exposure to marketing information rather than information about care services among consumers in the online community. In the context of the evidence and authority signals shown in Figure 4, while the FAQ provider group strives to enhance their credibility through research evidence, the online community’s response group portrays themselves as physicians or Korean traditional medicine practitioners and subtly mentions the hospitals where they practice, thereby attracting consumer interest.

### Limitations

This study had several limitations. First, the analysis used data from a single large Korean online community. Although this platform offers extensive information on ADHD, the findings may not comprehensively reflect the information needs of other communities, platforms, and cultural contexts. Second, although structural imbalances in existing FAQs were identified and directions for content restructuring were proposed, user-based evaluations are necessary to verify whether the revised information effectively meets consumer needs. Future work will involve restructuring health information and evaluating consumer satisfaction based on actual user assessment. Additionally, a pipeline should be established to systematically identify consumer health information needs.

### Conclusions

The ADHD FAQs created by institutional information providers covered half of the semantics of consumer questions posed in online communities. FAQs were concentrated within a limited number of FAQs, and many failed to address consumers’ unmet health needs. Twelve unmet needs were identified, offering a more comprehensive and detailed perspective than the current scope of information provided. Furthermore, these research findings indicate that using model-based similarity analysis and LLM-assisted analysis allows for accurate and scalable measurement of how well provider-generated FAQs address consumer questions. This methodology also aids in identifying significant gaps in the information provided. The restructured information produced through this approach is expected to enhance the clarity, accessibility, and relevance of FAQs, thereby more effectively addressing consumer needs.

## Supplementary material

10.2196/96060 Multimedia Appendix 1 Verification sample file for discrimination of similarity between frequently asked questions and community questions: samples of discrepancies and consistent opinions between reviewers.

## Abbreviations

ADHD: attention-deficit/hyperactivity disorder
FAQ: frequently asked question
LaBSE: Language-Agnostic BERT Sentence Embedding
LLM: large language model
MBERT: paraphrase-multilingual-MiniLM-L12-v2
mE5L: multilingual-E5-Large-instruct
Q&A: question and answer
RQ: research question
SBERT: Sentence-Bidirectional Encoder Representations from Transformers
