# Supplementary material for: What Provider Frequently Asked Questions Miss: Evaluating Unmet Attention-Deficit/Hyperactivity Disorder Information Needs Through Comparison of Online Community Posts Using Large Language Model–Assisted Semantic Analysis in a Mixed Methods Study
Source: J Med Internet Res. 2026 Sep 11;28:e96060. doi: 10.2196/96060 (PMC13564882; doi:10.2196/96060)
Supplement: Supplementary file 1 [file jmir-v28-e96060-s001.pdf]

First_Validation sample

A_idx	B_idx	A_text	B_text	label 1	label 2	final_label	Reviewr 1	Reviewer 2
18	1211	멍 때리는 우리 아이가 ADHD라구요?	남 초등 ADHD 어릴때 말을 좀 늦게 하긴 했는데 전체적으로 발달이 느린거 같은 느낌이 있어요. 다만 굉장히 산만해서 이제 초등학교1학년 올라가는데 학교 생활 잘 할 수 있을지 너무 걱정돼요. ADHD 진단 받고 치료 어떻게 받는지 문의해요. 아이가 잘 따라와주면 좋겠어요...	1	0	1	전체적으로 발달이 느린 부분에 대한 언급이 질문의 원본 내용과 겹치는 부분이 있고, 질문의 의도가 아이의 ADHD에 대한 걱정의 포인트 때문에 유사하다고 판단함.	A의 '멍 때리다'의 표현과 B의 '발달이 느리다'의 표현이 유사하다고 판단되어 수정함.
72	2179	ADHD 우리 아이의 숨겨진 강점	동생이 최근 성인 ADHD로 진단받아 치료를 시작할 예정입니다. 성인 ADHD의 치료가 전반적으로 어떤 식으로 진행되며, 완치 가능성이 있는지 알고 싶습니다. 치료 과정에서 어떤 점들을 유의하고 주의해야하는지, 일상 생활에서 도움이 될만한 방법은 무엇이 있을까요? ADHD 관련 치료와 관리에 대해 환자와 가족이 참고할 수 있는 종합적인 정보가 있으면 좋겠어요.	0	1	0	ADHD 아동의 창의성이나 유연성 등에 대한 강점에 대한 언급은 없고, 성인 ADHD에 대한 질문으로 유사하지 않다고 판단함.	A의 대상자 특성(아동)과 B의 대상자 특성(성인)이 확연하게 다르므로 수정함.
39	666	성인 ADHD에서 흔히 동반되는 진단	성인 ADHD 치료를 위해 약을 복용 중인데, 부작용이 걱정됩니다. 집중력은 나아지지만 가끔 소화 불량이나 불안감이 심해질 때가 있어요. 약물 부작용을 줄이기 위한 방법이 있나요? 아니면 약물 외에 증상을 관리할 수 있는 방법이 있는지 알고 싶습니다.	1	0	0	ADHD직접 연관질환은 아니지만, 치료과정 중 발생하는 소화불량과 불안감은 동반되는 질환으로 볼 수 있으며, 그에 따른 대처 방법을 묻는 질문이라고 생각하여 유사하다고 판단함.	A에서는 ADHD의 정신과적 동반진단(틱, 강박증)을 묻고 있는 것으로 보이나, B는 ADHD 치료제의 약물 부작용에 관해 묻고 있어 상이하다고 판단됨. 원안 유지함.
18	1406	멍 때리는 우리 아이가 ADHD라구요?	ADHD 아이가 과잉 행동을 증상으로 하는 ADHD가 있어서 고민 끝에 치료를 하려고 합니다. 아이가 받을 치료라서 치료 방법도 신중하게 결정하려고 하는데 ADHD 어떻게 치료하는 게 좋을까요?	0	1	0	아이가 멍때리는 부분에 대한 언급이 없고, A질문의 의도는 진단에 대해서, B질문에 대한 의도는 치료에 대해서 질문하므로 유사하지 않다고 판단함.	A와 B 모두 질문에 소아 ADHD 증상이 있어 범주가 다르더라도 동일한 답변이 가능할 것으로 판단했으나, 의도 해석이 주관적일 수 있어 수정함.
48	3289	성인 ADHD ? 대학생활 적응기	성인과 ADHD진단 비용 제가 고1 학생이고 입안 병원도 좋지않아요 부모님한테도 검사 받아보면 안되냐고 해봤지만 니가 무슨 ADHD 냐고 뭐라뭐라 욕먹었어요. 고1 되니까 대학과 취업문제가 확 다가오면서 이게 심각한 문제구나 생각이 들었어요. 진짜 학업에 너무 문제가 많아서 저 혼자라도 돈 모아서 병원 가봐야 할 거 같아요. 1. 상가에 있는 정신과를 갈건데요 ADHD 검사를 꼭 받아야 되는건가요? 그래야 ADHD라고 진단을 내릴 수 있는거예요? 이 검사가 몇 십 만원 이라고 들어서요. 2. 약물치료를 하고싶은데 부모님 동의서가 없으면 약을 절대 처방이 불가능인가요? 3. 돈이 최대한 적게 나올 수 있는 방법이 없을까요? 5~10만원 정도로요	1	0	0	고등학생이 ADHD증상과 관련하여 특히 학업에 대한 우려를 하고 있음. 원본질문과 관련하여 입시준비에 대한 부분도 포함되어 있으므로 검사와 치료에 대한 질문이지만, 답변은 거의 비슷한 내용으로 될것이라 생각하여 유사하다고 판단함	A의 대상자 특성은 성인, B의 대상자 특성은 청소년으로 확연히 다름. 또한 B의 질문 요지는 ADHD 진단 및 처방과 관련된 부분이라 A와는 답변 범주가 상이함. 원안 유지함.
63	2998	우리 아이의 ADHD를 어떻게 받아들이면 좋을까요?	아침에 일어나지 못 함(많이 심각. 사람이 깨워줘도 못 일어남. 일찍자도 못 일어남) 영상 배속으로 봄. 스킵함 집중을 못 함(한번에 집중하면 2시간이면 끝낼 일을 10분하고 딴짓 10분하고 딴짓 이래서 한 3-4시간 걸림) 청소 못 함(방이 더럽다 많이) 까먹음. 맨날 잃어버림(물건을 못 챙김)(잃어버려서 같은 걸 새로 사고 또 새로사고 하다 결국 나중에 같은 물건이 3-4개 생김. 근데 그러다 또 다 잃어버려서 다시 사고 무한반복) 걱정?그런게 많음. 멍을 자주 때림 머리가 쉬지를 않는 것 같다(계속계속 상상?생각?하는듯) 할 일이 많으면 정리가 안됨(이거 하다가 중간에 저거하고 또 저는 모든 사람이 다 이런 줄 알았는데 ADHD라는 걸 알고나서 제가 문제거 있는건가 하는 생각이 들었어요 아시는 분은 어때 보이는지 알려주시요 .....ㅠ	1	0	0	여러가지 증상들을 약간을 지리멸렬하게 늘어놓았지만, 결국은 본인의 ADHD에 대한 상태를 직면하고 받아들이는 부분에 대한 혼란이 있는것으로 보아 유사하다고 판단함	A는 부모 측면에서, B는 본인 측면에서 ADHD의 수용 및 관리법을 질문하는 것으로 판단됨. 대상자의 특성이 확연이 달라 답변 범주가 상이할 것으로 판단됨. 원안 유지함.
50	4495	ADHD 치료를 하지 않으면 후유증이 남을까요?	제가 어렸을 때부터 산만하고 집중력이 부족했는데 그게 ADHD 증상이라는 걸 뒤늦게 알았습니다. 찾아보니 어릴 때 ADHD가 있으면 치료를 받는 것 같은데 성인 ADHD도 치료를 하는지 그리고 치료를 받으면 좋아질 수 있는지 궁금해서 문의드립니다.	1	0	1	어릴 때 ADHD 증상을 인식했지만, 뒤늦게 알아차려 성인 ADHD인것 같다고 생각함. 그에 따른 걱정과 치료에 대한 의문이므로, 치료를 하지 않아서 발생하는 후유증에 대한 질문과 유사하다고 판단함.	피드백 의견에 동의하여 수정함.
60	2204	ADHD, 진단과 경계 너머의 이야기/ 종종 약속을 잊거나 물건을 잃어버리고 충동적으로 물건을 구매하는 나 ADHD일까요?	늘 불안하고 집중안되고 직장 자주 옮기게 되는데 제가 혹시 ADHD성인 해당할까요?	1	0	1	증상이 약간 다르기는 하나, 불안과 집중, 이직에 대한 부분이 ADHD 증상으로 인한 진단에 대한 의문이므로 유사하다고 판단함.	A와 B에서 언급된 증상이 서로 다르나, 결국은 모두 ADHD로 귀결되어 동일한 답변이 가능할 것으로 판단됨. 수정함.
73	2092	성인 ADHD의 계획 세우기	성인ADHD 저도 어릴 때 산만하다는 말을 많이 들었는데, 지금은 직장생활에서 집중이 안 되고 일이 자꾸 밀립니다. 혹시 성인ADHD일까요?	1	0	1	성인 ADHD에 대한 질문이라는 점과, 집중이 안되고 일이 밀린다는 부분에 대한 언급으로 보아, 계획세우기와 유사하다고 생각되어 유사하다고 판단함.	피드백 의견에 동의하여 수정함.
17	756	사회기술훈련을 하면 ADHD가 좋아지나요?	ADHD 안녕하세요, 저희 아이는 올해 초등학교 1학년인데요, 학교에 들어가면서부터 집중력, 행동 문제로 어려움을 겪고 있어요. 심한건 아니지만.. 학기 시작하고 1달 다 되어가는데 아직도 한번씩 수업시간에 돌아다니고 친구들을 방해한다고 해요. 제가 관찰해보니 숙제도 혼자 잘 하지 못하고 자꾸만 산만해지는 것 같아요. 인터넷으로 찾아보니 ADHD 증상과 비슷하다고 하는데, 정말 ADHD일까요? 제가 어떻게 해줘야 아이가 잘 자랄 수 있을지 고민이 많습니다. 도움을 부탁드려요.	1	0	0	학교생활에서의 어려움과 관련하여 아이가 잘 자랄 수 있을지에 대한 의문을 가지고 있는 질문으로, 사회기술훈련이라는 용어를 모르지만 얻고자 하는 답변의 내용은 비슷할것이라고 생각하여 유사하다고 판단함	ADHD는 병리적 질환으로 의학적 진단 및 관리(투약 등)의 병행이 중요함. B 질문에 대한 1차적 답변으로 비의학적 처치(사회기술훈련)이 먼저 도출되는 것은 바람직하지 않다고 생각함. 원안 유지함.
43	935	우리아이, 자존감과 사회성의 관계 : 나를 가치롭게 여기는 마음 '자존감', 남들과 잘 어울리는 능력 '사회성'	adhd 아이 adhd 증상 때문에 고민이예요.. 초등 3학년 자녀 때문에 정말 고민이 많아요. 작년부터 계속 집중을 못하고 수업시간에 자꾸 딴짓하고 친구들이랑도 자주 트러블이 생겨서 담임선생님께서 상담을 권하셨어요. 증상을 보면 숙제할 때 5분도 못 앉아있고, TV나 게임할 때만 집중하고, 물건도 자주 잃어버리고, 말도 끝까지 안 듣고 튀어나갑니다. 이런 것이 벌써 1년 넘게 계속되고 있어요. 몇 달 전에 소아정신과에서 검사도 받아봤는데 adhd 성향이 있다고 들었습니다. 약물 치료를 권했으나 아직 어린 나이에 약을 먹이는 게 걱정돼서 다른 방법이 없을지 알아보고 있습니다. 혹시 한의원에서도 이런 아이들을 진료할 수 있는지, 인천 지역에서 한방 소아과나 신경정신과 진료가 가능한지 궁금합니다. 침이나 한약도 도움이 될 수 있을까요? 자녀가 점점 자신감도 없어하고 학교 가기 싫어해서 부모로서 마음이 아픕니다. 어떻게 해야 할지 막막합니다	1	0	0	초등학생의 사회에서의 어려움과, 아동의 자신감이 없는 상황과 관련된 부모의 걱정에 대한 답변을 얻기를 원하는 질문으로, 동일한 의미로 판단함.	B의 질문 요지는 인천 지역 치료법(신경정신과 진료, 침, 한약 등)에 대해 묻는 것으로 A를 통해 도출되는 답변과는 범주가 상이할 것으로 판담됨. 원안 유지함.
32	701	우울한데, ADHD?	ADHD 제가 adhd 진단을 받았는데 어린 나이일 때는 제가 adhd인 줄 몰랐는데 이제보니 adhd 맞는 것 같아서요. 성인과 어린이의 adhd 증상 차이는 큰가요?	0	0	0		
25	1730	아이가 알약을 못 삼키는데, ADHD약은 가루약이 없나요?	ADHD 아이가 집중력이 너무 떨리고 말을 잘 못알아듣는 경향이 있어요 이 외에도 걱정스런 부분들이 많아서 ADHD같은데 혹시 ADHD테스트하는 방법있을까요? ADHD일때 나오는 증상이나 특정행동같은게 있는지요 한의원치료도 궁금해요	0	0	0		
7	3426	어린시절 ADHD 진단을 받은 사람만 성인 ADHD인가요?	제가 성인 ADHD인 것 같아서 진단과 상담, 필요하다면 약물 처방까지 받아보고자 합니다 이때 1) 상담 치료만으로도 기록이 남는지, 2) 약물 처방 기록이 남아있다면 추후 문제가 될 수 있는지 궁금해서 질문드립니다	0	0	0		
6	2406	성인 ADHD는 치료를 받으면 완치가 가능한가요?	집중이 잘 안되고 뭘 하려고 할때까지 시간이 너무 많이 걸려요 해도 몇 분하고 또 딴짓하고... adhd인가요...	0	0	0		
32	2854	우울한데, ADHD?	ADHD인가요 집중을 3분이상 해본적없고 학원에서 수업 들을때마다 설명 그만했으면 좋겠고 몸치이고요 다리가 간지럽고 정문으로 뛰어내리고싶습니다 어릴때부터 집중을 못 했고 공부할때마다 이렇게 가 같아서 성적도 바닥인데 이거 해결방도 없습니까	0	0	0		
56	254	아이도 힘들지만 부모인 나도 힘들다 (ADHD부모교육 및 부모상담) "선생님 아이가 ADHD를 진단받고 저 자신도 너무 힘들었어요. 아이를 어떻게 다루어야 할지도 모르겠는데 주변에서는 다들 부모가 잘못 키웠다며 비난을 하니 제가 우울증 약을 먹어야 할 것 같아요"	ADHD 원래 수험 준비할 때부터 강의에 집중을 못하고 주변 정리 정돈도 못했습니다 기억력도 안 좋고요, 지금은 대학생인데 물건을 자주 잃어버리고 제 스스로도 산만하다는 느낌이 듭니다. 인터넷으로 ADHD 테스트를 해봤는데 점수가 높게 나오더라고요. 앞으로 시험 준비도 남아있고 사회생활도 해야 되는데 걱정이 많이 됩니다. 신경정신과 한의원에서 부작용 없이 성인 ADHD 치료를 받을 수 있을까요?	0	0	0		
60	2665	ADHD, 진단과 경계 너머의 이야기/ 종종 약속을 잊거나 물건을 잃어버리고 충동적으로 물건을 구매하는 나 ADHD일까요?	ADHD가 아닌가 하는 의심은 성인이 되고 나서야 들기 시작했는데요. 초등학교 입학 때부터 꼭 한 명씩 지각하던 게 중고등학교 들어서며 개선되고 성인이 된 이후로는 거의 하지 않게 됐어요. 대신 평소에 이것저것 자꾸 잊어버립니다. 스스로도 이상하게 여겨질 정도로 심부름 받은 걸 까먹은 적도 많고, 빨래 돌려놓고 까먹어서 다시 돌릴 때도 많아요. USB같은 작은 물건은 자주 깜빡하고요. 중학생 때까지는 과목 수업에서 낙서하거나 서로 분해하면서 만지하는 게 일상이었어요. 대화하는 중에 상대방이 무슨 말을 하는지 잘 못 듣고요. 말하는 중에 끼어든다던가 못 기다리는 일은 없는데 검사 받아보는 게 좋을까요?	1	1	1		
45	403	예민한 기질에 감춰진 ADHD?	ADHD 아이가 열여 전 ADHD 진단을 받았습니다. 증상은 주의 집중력 부족인데 수업 시간에 집중을 못 하고 산만하게 행동해서 다른 친구들에게도 방해가 돼서요. 이 경우 어떻게 치료하는 게 좋을까요?	0	0	0		
55	1737	산만한 우리아이, 아직 너무 어린데 ADHD일까요?	아이가 ADHD 진단을 받았습니다. 증상으로 충동성이 두드러지게 나타나는데 이거 때문에 다른 사람들에게 안 좋게 보일까 봐 신경도 쓰이고 걱정도 됩니다. 그래서 초기에 치료하려고 하는데 치료를 받으면 ADHD 좋아질 수 있을까요?	0	0	0		
83	3049	디지털 시대와 ADHD:소셜미디어와 게임이 ADHD에 미치는 영향	Adhd의 증상중 집중못하고 핸드폰 자주 만지는것도 포함되나요???	1	1	1		
58	1752	ADHD, 그 다양한 모습: 외현화 증상과 내재화 증상	ADHD 유치원에서 거짓말도 종종 하고 장난감도 집으로 가지고 올때도 있어요 산만함도 심하고 에너지가 넘쳐요. 에너지가 넘치는건 돌 지나서부터 많이 심했고 호기심이 많아서 어른들이 지칠 정도로 끊임없이 질문을 많이 합니다. 근데 질문이 정말 어른들이 말하기 곤란한 질문을 해요 작년에는 훈육이 힘들어 상담센터 다녔는데 ADHD확률이 높다고 큰 병원 가서 검사 받아 보라세요 집에서는 사실 큰 문제가 없어 보이는데 유치원에선 지적이 많아요. 근데 제 생각엔 ADHD 검사에서 확실히 맞다 안맞다기도 어린나이이셔 병원마다 검사 결과가 다르다는 얘기도 많이 들었어요. 아이는 어릴 때부터 많이 산만했고 호기심도 많았는데 성향 차제가 그런 것 같다는 느낌도 듭니다.	1	1	1		
70	1084	ADHD라면서 왜 다른 약을 주세요?	ADHD 아이의 ADHD 치료를 한의원에서 받으려고 하는데 ADHD 치료에 한약, 침치료 어떤가요? 치료 내용이 궁금한데 자세히 알려주실 수 있을까요?	0	0	0		
63	3256	우리 아이의 ADHD를 어떻게 받아들이면 좋을까요?	안녕하세요. 초등학교 2학년 아들이 요즘 수업시간에 집중하지 못하고, 친구들에게 장난치는 빈도가 높아서 걱정이 돼서 병원을 다녀왔습니다. 병원에서 ADHD 진단을 받았고, 의사 선생님께서 약물치료를 권장하시더라고요. 아직 아이가 어리다 보니 약물치료가 정말 필요한지도 그렇고 부작용은 없는지 걱정이 큽니다. 약물치료 외에 다른 방법은 없는지도 궁금하고요. 비슷한 상황을 겪으셨거나 조언해주실 분들이 계시면 도움 부탁드립니다. 어떻게 하는 게 아이에게 가장 좋은 선택일까요?	1	1	1		
32	4549	우울한데, ADHD?	adhd 요즘 어른들 보면 adhd가 있다고 하잖아요. 지금 제가 집중력 떨어지고 그런 거 보면 adhd인 거 같은데, 치료 안 받으면 성인기까지 이어지나요? 아니면 치료를 받고도 안 나아서 성인까지 이어지는 건가요? 어린이 adhd는 어떻게 치료하는 것이 좋아 되나요?	0	0	0		

Second_Validation sample

A_idx	B_idx	A_text	B_text	label 1	label 2	final_label	Reviewr 1	Reviewer 2
20	288	학교에서는 ADHD 학생을 어떻게 도와주어야 하나요?	저는 현재 고1 학생입니다.제가 요즘에 학교에서 집중을 잘 못하는것 같아 찾아보니까 adhd 증상과 비슷하더라고요. 아무튼 집중이 안돼서 화가 나고 결국 항상 제 시간 이내로 풀지 못합니다..그래서 모고 국어는 5등급 위로 올라간 적이 한 번도 없어요.또한 제가 생각해도 남들보다 제가 말귀를 잘 못 알아먹는 것 같습니다.근데 제가 보편적인 adhd보다,보통 애들보다 말수가 엄청나게 적습니다. 조용한 adhd의심 해봐야 되는걸까요?	0	1	0	현재 작성자가 궁금해하는 것은 본인의 증상을 나열하고 이에 대해 조용한 ADHD 에 해당하는지 여부입니다. 학교 입장에서는 고려가 되지않아 유사하지 않다고 판단함.	피드백 의견에 동의하여 수정함.
48	2232	성인 ADHD ? 대학생활 적응기	최근 일상에서 집중이 어렵고, 실수가 잦아져서 걱정이 많아졌어요. 회사 업무 중에도 자꾸 다른 생각이 들거나, 물건을 자주 잃어버리곤 합니다. 이런 증상들이 혹시 성인 ADHD와 관련이 있을 수 있을까요? 성인 ADHD는 아이들과 어떻게 다른 특징을 보이나요? 주의력 저하 외에 감정 조절이나 대인관계에서도 문제가 나타날 수 있나요? 이런 증상을 겪고 있다면 어떤 검진이나 상담을 받아야 할까요?	0	1	0	회사업무' 중에라는 표현으로 대학생활과 는 조금 거리가 있고, 성인 ADHD와 아동의 ADHD차이를 묻고, 진단검사에 대해 궁금증이 포커싱되어있어서 유사하지 않다고 판단함.	대학생 중에서도 인턴 및 기간제 근로를 통해 '회사' 업무를 경험할 수 있을 것 같아 '1'로 판단함. 다만 질문의 요지 측면에서 A-B 불일치성이 크다는 피드백을 수용해 수정함.
6	2340	성인 ADHD는 치료를 받으면 완치가 가능한가요?	최근에 성인 ADHD 진단을 받아 다음 진료 이후부터 약물치료를 해보기로 했어요. ADHD 약을 복용하면 효과가 언제부터 나타나는지 궁금합니다. 약효가 나타나기 시작하면 보통 어떤 변화를 가장 크게 느낄 수 있나요? 약을 먹기 시작하면 보통 얼마나 오래 복용하는지, 내성이나 부작용은 없는지도 궁금합니다.	1	0	0	성인 ADHD로 명시하였고, 약물복용의 결과, 약을 먹는 기간과 부작용 등이 치료의 종결에 대한 궁금증도 포함되어 있다고 판단하여 유사하다고 판단함.	A의 질문은 치료의 '종결' 시점에 관해, B의 질문은 치료의 '시작' 시점에 관해 초점이 맞춰져 있음. B의 질문 일부가 A 질문과 겹치지만 동일한 답변이 도출될 경우 B의 궁금증 대다수는 해결하기 어려움. 따라서 원안 유지함.
39	616	성인 ADHD에서 흔히 동반되는 진단	남 성인ADHD 예전부터 집중못하고 산만함 불안하고 우울해요 20대가 된 지금도 계속... 성인ADHD 증상이라고 하던데 성인ADHD 치료 어찌하죠	1	0	1	성인 ADHD로 불안과 우울의 증상이 동반되는 진단이라고 스스로 판정하고 그에 대한 치료방법을 묻는것으로 생가되어 유사하다고 판단함. A_text의 원문을 통해서 동일한 답변이 될 수 있다고 생각함.	피드백 의견에 동의하여 수정함.
58	1569	ADHD, 그 다양한 모습: 외현화 증상과 내재화 증상	ADHD 20대 여자입니다. 제가 약속과 해야 될 일을 자주 잊습니다. 물건도 자주 잃어버리고 직장 생활을 하고 있는데 업무에도 지장이 갈 만큼 집중력도 떨어집니다. 대화를 할 때도 집중을 못 해서 중간에 다른 생각이 계속 들어요. 인터넷에서 ADHD 테스트를 해봤는데 거의 다 해당이 되더라고요 성인 ADHD도 정신과 한의원에서 치료가 가능하나요?	0	1	0	다양한 증상에 대해서 언급하였지만, 질문의 요지는 이러한 증상을 한의원에서 치료가 가능한지를 묻는 질문으로 유사하지 않다고 판단함.	피드백 의견에 동의하여 수정함.
12	1469	약을 얼마나 오래 먹어야 하나요?	남 ADHD 아이가 수업 시간에 집중을 못 하고 산만한 행동을 계속 보인다는 이야기를 들었습니다. ADHD가 아닐까 하는 의심이 있었는데 담임 선생님께 연락을 받은 후 그냥 방치하면 안 되겠다는 생각이 들더라고요. 우선 제대로 검사를 해보려고 하는데 ADHD라고 한다면 어떻게 치료하는 게 좋을까요?	0	1	1	약물치료에 대한 언급이 빠져있고 일반적인 ADHD치료방법에 대한 질문으로 유사하지 않다고 판단함.	ADHD는 병리적 질환이므로 치료의 제1원칙은 '약물치료'임. 행동치료를 비롯한 비약물치료는 보조적인 치료로 판단됨. 따라서 A와 B 질문에 대한 답변 모두 동일한 범주에서 다룰 수 있다고 생각함. 따라서 원안 유지함.
82	2117	집중력 약을 먹여도 될까요?	남 성인ADHD 일할때 집중안되고 자꾸 못한다고 듣는데 성인ADHD 병원 가봐야 하나요	0	1	1	성인 ADHD 진단이나 치료를 위한 병원방문에 대한 의견을 묻는 질문으로 집중력 약으로 한계짓기는 어려워서 유사하지 않다고 판단함.	ADHD는 병리적 질환이므로 치료의 제1원칙은 '약물치료'임. 행동치료를 비롯한 비약물치료는 보조적인 치료로 판단됨. 따라서 A와 B 질문에 대한 답변 모두 동일한 범주에서 다룰 수 있다고 생각함. 따라서 원안 유지함.
30	1613	알기 쉬운 성인 ADHD	30대 초반/여 ADHD 안녕하세요. 우리 아이가 ADHD 진단을 받았는데, 충동 조절이 되지 않아 심한 말다툼이나 때리기와 같은 폭력적 행동이 자주 나타납니다. 친구를 밀치거나 사소한 일에도 소리를 지르며 화를 주체하지 못해 학교와 가정 모두가 긴장 상태예요. 이런 과도한 공격성을 어떻게 이해하고 관리해야 할지 막막합니다.	1	0	0	ADHD를 진단받은 성인으로 자신에게 나타나는 증상을 어떻게 받아들여야 하는지에 대한 질문으로 '알기쉬운 성인 ADHD'의 질문의 세부내용을 살펴보면 자신을 이해하는데 도움이 될것이라고 생각하고, 성인 ADHD증상에 대한 추가적인 정보를 요청하는 의도로 파악되어 유사하다고 판단함.	B의 질문자는 30대 성인 여성이지만, 질문 내용은 본인의 아이(소아)의 증상 및 관리 방법에 대해 묻고 있음. 따라서 A와 B는 서로 상이하다고 판단됨. 원안 유지함.
56	1061	아이도 힘들지만 부모인 나도 힘들다<br><br>(ADHD부모교육 및 부모상담) "선생님 아이가 ADHD를 진단받고 저 자신도 너무 힘들었어요. 아이를 어떻게 다루어야 할지도 모르겠는데 주변에서는 다들 부모가 잘못 키웠다고 비난을 하니 제가 우울증 약을 먹어야 할 것 같아요"?	남 ADHD 안녕하세요 아이 소아 ADHD 치료 걱정되고 답답하네요 집중못하고 산만하고 잠시도 가만히 안있으려하고요 커가면서 나아지겠거니 했는데 어떻게 해야 하나요??	1	0	1	ADHD를 키우는 부모로써 자녀를 어떻게 다루어야 할지에 대한 고민이 포함된 질문으로, 동일한 답변을 제공할 수 있다고 생각하여 유사하다고 판단함.	피드백 의견에 동의하여 수정함.
60	2293	ADHD, 진단과 경계 너머의 이야기/ 종종 약속을 잊거나 물건을 잃어버리고 충동적으로 물건을 구매하는 나 ADHD일까요?	인터넷에 돌아다니는 성인 ADHD 자가진단 항목을 체크해보니,딱 제 얘기 같아서요ㅠ 산만하다, 정신없다 이런 소리는 종종 듣지만, 그래도 공부도 꽤 잘했던 편이고해서 제가 ADHD일거라는 생각은 전혀 못했거든요. 오히려 정기적으로 좀 우울해지고 다운되는 경우가 있어서 조울증인가 의심하기도 했었고요. 정확히 성인 ADHD인지 알 수 있는 검사나 치료를 받을 수 있는 곳 있으면 알려주세요!	0	1	0	질문의 의도는 ADHD의 진단가능성을 고려하여 실제적으로 검사나 치료를 위한 병원에 대한 정보를 요청하는 질문으로 유사하지 않다고 판단함.	피드백 의견에 동의하여 수정함.
5	2711	소아 ADHD와 성인 ADHD증상이 다른가요?	ADHD 중학생이 되고나서 유난히 집중을 못하는 것 같습니다. 물건도 자주 잃어버리고, 불러도 멍할 때가 자주 있어요. 공부를 잘 하지는 못해도 그래도 할일은 하는 편이었는데, 이상하게 이전보다 의욕도 없는것 같고, 집중을 못하는데, ADHD일까요? 제가 성인ADHD라서 아이도 닮은 게 아닐까 걱정이 됩니다.	1	0	1	ADHD가 의심되는 자녀에 대한 증상을 나열한 뒤, 자신의 ADHD진단을 언급하며서 그것을 닮아서 그런것인지, 그래서 자녀가 ADHD인지에 대한 궁금증을 가지고 있음. 따라서 소아와 성인의 증상 차이에 대한 궁금증으로 판단하여 유사하다고 판단함.	피드백 의견에 동의하여 수정함.
44	727	청소년기에 진단된 주의력결핍 과잉행동장애 청소년 자녀,<br><br>집중을 잘 못 하는데, 혹시...	ADHD 저는 30대 식상인입니다. 제가 10년 선부터인거 같은데 스스로 산만함이 느껴질 정도로 집중력이 부족하고 가만히 있지를 못합니다. 대화를 할 때도 다른 생각을 할 때가 많고요. 영화나 드라마를 볼 때도 내용에 집중을 못 하고 멍하게 있는 경우가 많습니다. 집중력 부족으로 업무를 제시간에 끝내기도 힘들고요.. 병원에서 성인ADHD 진단을 받고 2년째 양약을 복용 중인데 조금 나아진 듯하더니 최근들어 증상이 더 심해진 느낌이 들어서요.. 주위에서 신경정신과 한의원 치료를 권유해서 문의드립니다. 성인 ADHD도 치료가 가능할까요? 치료 기간은 어느정도로 생각하면 될까요 ?	0	1	0	성인 ADHD로 자녀와는 관련성이 떨어지고, 자신의 증상에 대한 치료방법에 대해 묻는 질문으로 유사하지 않다고 판단함.	B의 질문자의 현재 연령(30대)에서 10년 전을 역산했을 때(20대) 후기 청소년기에 해당할 수 있을 것 같아 '1'로 판단함. 다만 A는 대상 범주가 청소년 '자녀'이므로 상이한 것으로 판단하는 것이 적절해 보임. 따라서 수정함.
48	2396	성인 ADHD ? 대학생활 적응기	제가 성인 ADHD가 맞을까요? 저는 고등학교 3학년 학생입니다. 유튜브 영상 하나를 봤는데 ADHD증상이 저와 너무 똑같이 겹치고 마침 저도 그러한 이유들로 스트레스를 받고있었어서 그게 혹시 제가 ADHD여서 그런걸까 싶어서 검사를 받아보려고 하는데요 다름이 아니라 ADHD는 후천적으로 발병하는 질환이 아니라고 들어서요.. 제가 어렸을때 약간 산만하다는 소리는 들어봤어도 막 ADHD 검사 받아봐라 이런 소리는 못들어봤는데 ADHD가 어릴때 티가 안날 수가 있나요? 지금 당장 성인ADHD증상으로만 봤을때는 거의 모든증상이 저에게 나타나고있거든요	0	1	0	고등학교 3학년의 ADHD의심에 대한 질문으로 학업이나 대학생활과 관련된 내용이 문장에 언급되어 있지 않아 유사하지 않다고 판단함.	피드백 의견에 동의하여 수정함.
72	2896	ADHD 우리 아이의 숨겨진 강점	안녕하세요 고2 학생입니다 진짜 한 중3 때 부터 가만히 있다가 갑자기 급발진해서 뛰어다니고 또 흥분하면 가만히 못 있겠어요 그리고 과제 할 때나 수업 들을 때도 계속 딴 생각나고, 수행으로 보고서 같은거 작성 할 때도 하나에 몰두하면서 생각하는게 잘 안 됩니다ㅠㅠ 근데 또 수학, 과학 같은 문제 풀땐 집중이 되긴해서 좀 애매해요 저 조용한 adhd인가요? 저 병원가야되나요?	0	1	0	강점이 살짝 언급되긴하였지만, 자신이 조용한 ADHD에 대한 진단가능성이 있는지에 대한 질문으로 강점과 관련된 콘텐츠와 맞지 않다고 생각되어 유사하지 않다고 판단함.	피드백 의견에 동의하여 수정함.
0	2281	ADHD의 주요증상은 무엇인가요?	최근 집중력이 떨어져서 일상생활에 어려움을 겪고 있습니다. 성인 ADHD 진단에 풀배터리 검사가 효과적인가요? 이 검사를 통해 어떤 증상들이 확인될 수 있나요? 진단 후 치료 방법은 어떻게 달라질까요? 풀배터리 검사 결과는 얼마나 신뢰할 수 있는지 궁금합니다.	0	0	0		
0	886	ADHD의 주요증상은 무엇인가요?	ADHD 티비를 보다가 저도 성인 ADHD가 아닐까 생각을 했는데 검사를 해보니 정말 그렇다고 하네요 아이가 아닌 성인 ADHD도 치료하면 좋아질 수 있을까요?	0	0	0		
1	3339	ADHD는 얼마 동안 약을 먹어야 할까요?	제가 지금은 부담임 하고 있고 내년엔 정담임 하려고 했는데 실생활에 너므 부족한게 많아서 ADHD 검사를 했는데 약물 처방 권유 받았어요... 정담임은 되면 학부모와 직접 대면하는 것도 그렇고 할일이 많을텐데 벌써부터 걱정이에요... 사실 대학교에서 배웠던 거 기억도 잘 안나고요... 어떻게 하면 좋을까요	0	0	0		
1	1980	ADHD는 얼마 동안 약을 먹어야 할까요?	아이가 너무 산만한데 adhd인지 알고 싶어요 집중할 때는 하는 것 같기도 해서 adhd가 맞는지 아닌지 헷갈리네요 adhd를 알 수 있는 검사가 있나요? ADHD라면 빨리 고쳐주고 싶네요 adhd라면 어떻게 치료해야하죠? adhd원인이 뭔가요? adhd아이에게 좋지 않은 게 있으면 알려주세요~	0	0	0		
1	2142	ADHD는 얼마 동안 약을 먹어야 할까요?	20대 ADHD 주의력결핍인것같아 걱정되어 고민입니다. 만약에 그렇다면 무조건 성인adhd로 진단받나요? 어릴때도 그랬던것같은데 생각해보면 흑역사같아요.. 맨날 저만 뛰어다니고 갑자기 돌아다닌다고 혼났고..주의받고 벌점받았던 과거의 기억이 떠올라요. 내가 정말 adhd가 아니였나 하는 생각에 슬퍼지네요. 그리고 카드값도 정산해보면 너무 충동적으로 불필요하게 산게 많아서.. 집에 있는데 또 사고.. 미리 쟁겨놓는 습관도 고치고 싶어요. 정신과약을 먹고 오히려 다른 부작용을 겪을 까봐 걱정인데 다른 진료나 치료방법이 있는지 좀 알려주세요.	0	0	0		
2	3116	ADHD 약을 복용하면 머리가 좋아지나요?	ADHD 진단을 받으면 회사나 학교에 꼭 알려야 하나요? 진단을 받아도 그걸 굳이 오픈하지 않아도 된다고는 하는데, 배려를 받으려면 말해야 한다는 말도 들었어요. 근데 편견이나 차별이 생길까 봐 말하는 게 두렵기도 하더라고요. 장단점이 분명 있을 것 같아서요. 혼자 감당하기엔 벅찬데 말한다고 해서 이해받을 수 있을지도 모르겠고요. 공개 여부를 어떻게 결정해야 할지 고민입니다.	0	0	0		
2	1017	ADHD 약을 복용하면 머리가 좋아지나요?	ADHD 얼마 전 ADHD 진단을 받았습니다. 제대로 치료받고 싶은데 어떻게 치료하면 좋을까요?	0	0	0		
2	589	ADHD 약을 복용하면 머리가 좋아지나요?	ADHD 증상 테스트를했는데 전혀 아니라고 나옵니다 남들이 보았을때 그리보인다면 ADHD인가요?	0	0	0		
3	1231	부모가 ADHD였다면 자녀도 ADHD일 확률이 높나요?	ADHD ADHD 때문인지 오랜 시간 집중하지 못하고 계속 중간중간 딴짓을 하게 됩니다. 다른 사람이 말을 해도 정확하게 이해하기 힘들고 쓸데없는 행동을 자주 하게 돼요. 이런 ADHD 증상은 어떻게 치료해야 하나요?	0	0	0		
3	3222	부모가 ADHD였다면 자녀도 ADHD일 확률이 높나요?	ADHD 올해 8세가 된 딸이 가만히 있지 못하고 남자아이들처럼 산만하고 과격한 행동이 보여서요. 학교 선생님 권유로 소아정신과에 갔더니 ADHD 진단을 받았습니다. 의사선생님 조언에 따라 약을 복용시켜봤는데 밤에 잠을 못 자고 토를 자주 하더라고요 아무래도 ADHD약물 부작용인 것 같아서 성장중인 아이에게 방해가 될까 봐 약을 중단시켰는데 치료가 된 상태가 아니어서 여전히 산만함을 그대로입니다. 치료방법을 바꿔 한방치료는 어떨까 하는데 소아 ADHD도 치료가 될까요? 부작용과 재발 없는 치료를 원합니다	0	0	0		
